# Radio-resistant mesenchymal stem cells: mechanisms of resistance and potential implications for the clinic

**DOI:** 10.18632/oncotarget.4358

**Published:** 2015-06-08

**Authors:** Nils H. Nicolay, Ramon Lopez Perez, Rainer Saffrich, Peter E. Huber

**Affiliations:** ^1^ Department of Radiation Oncology, Heidelberg University Hospital, Heidelberg, Germany; ^2^ Heidelberg Institute for Radiation Oncology (HIRO), National Center for Radiation Research in Oncology, Heidelberg, Germany; ^3^ Department of Molecular and Radiation Oncology, German Cancer Research Center (dkfz), Heidelberg, Germany; ^4^ Department of Hematology and Oncology, Heidelberg University Hospital, Heidelberg, Germany

**Keywords:** mesenchymal stem cell, radiotherapy, double strand break, tissue regeneration

## Abstract

Mesenchymal stem cells (MSCs) comprise a heterogeneous population of multipotent stromal cells and can be isolated from various tissues and organs. Due to their regenerative potential, they have been subject to intense research efforts, and they may provide an efficient means for treating radiation-induced tissue damage. MSCs are relatively resistant to ionizing radiation and retain their stem cell characteristics even after high radiation doses. The underlying mechanisms for the observed MSC radioresistance have been extensively studied and may involve efficient DNA damage recognition, double strand break repair and evasion of apoptosis. Here, we present a concise review of the published scientific data on the radiobiological features of MSCs. The involvement of different DNA damage recognition and repair pathways in the creation of a radioresistant MSC phenotype is outlined, and the roles of apoptosis, senescence and autophagy regarding the reported radioresistance are summarized. Finally, potential influences of the radioresistant MSCs for the clinic are discussed with respect to the repair and radioprotection of irradiated tissues.

## INTRODUCTION

Mesenchymal stem cells (MSCs) were first characterized by Friedenstein et al. in 1974 after isolation from mouse bone marrow [1]; they were initially described as colony-forming, spindle-shaped cells, able to rapidly adhere to plastic surfaces and differentiate into adipocytes, chondrocytes and fibroblasts. Unlike their bone marrow-derived hematopoietic counterparts, MSCs form heterogeneous populations; to account for this fact and to differentiate them from embryonic stem cells, they are sometimes termed multipotent mesenchymal stromal cells [2, 3]. MSCs express various cell surface proteins, including CD13, CD29/ITGB1, CD44, CD73, CD90, CD105/ENG and CD106/VCAM-1 and lack hematopoietic surface markers CD31, CD34, CD45 and CD116 [4, 5]. However, a unique pattern of surface markers has yet to be generally accepted to prospectively identify MSCs; therefore, functional aspects such as the cells' fibroblast-like appearance, their ability to rapidly adhere to plastic surfaces and form colonies in culture and their potential for differentiation along the osteogenic, chondrogenic and adipogenic lineage are commonly employed to further characterize MSCs [6]. A combination of functional characteristics and molecular marker expression patterns has been suggested as minimal criteria for defining multipotent MSCs [7]. Beyond their appearance in the bone marrow, MSCs have been detected and isolated from a broad variety of other tissue types including vascular, adipose and skin tissues, kidney, umbilical cord and placenta [6, 8-10].

As MSCs were first isolated from bone marrow, their physiological function has been most widely studied in the context of this organ. The adult bone marrow contains the key lineages of hematopoietic stem cells (HSCs) and maintains and proliferates them according to demand [11]. HSCs are kept in a specialized microenvironment, termed the HSC niche, containing various other supporting cell types, including MSCs [12-14]. These supporting cells have been shown to secret extracellular matrix proteins such as VCAM-1, N-cadherin or annexin II, providing a scaffold for HSC retention [15, 16]. Additionally, the regulation of HSC proliferation, mobilization or differentiation is intricately controlled within the stem cell niche by the secretion of different signaling molecules, among them growth factors, chemokines and cytokines [17, 18]. MSCs have been among the most well-characterized supporting cells in the HSC niche, and similar functions regarding organ-specific cellular homeostasis have been attributed to MSCs based in lung tissue or blood vessels [19, 20].

Despite the fact that the physiological roles of MSCs are only incompletely understood, these cells have been subject to intensive research in recent years, as they may hold the potential to participate in the repair of tissue damage. Animal studies have shown their ability to migrate and integrate into damaged organs and differentiate into organ-specific, functional cells [21, 22]. Additionally, MSCs may create a protective and nurturing microenvironment inside damaged tissue by secreting a multitude of growth factors and cytokines, among them stem cell factor (SCF), vascular endothelial growth factor (VEGF), basic fibroblast growth factor (bFGF), hepatocyte growth factor (HGF), angiopoietin, platelet-derived growth factor (PDGF), transforming growth factor β (TGF-β), nerve growth factor (NGF) and brain-derived neurotrophic factor (BDNF), thereby aiding other cells in the regeneration of damage [23-32]. The regenerative potential of MSCs has widely been shown *in vitro* and in animal models, and the beneficial effects of MSCs have been discussed for various forms of tissue damage, including myocardial scars, cartilage injuries, pulmonary damage as well as skin and nerve tissue defects [33-35].

While the regenerative properties of MSCs have been well examined in the context of ischemic or mechanic tissue lesions, these effects may also be applicable to other forms of damage, especially the detrimental effects of ionizing radiation on treated or exposed normal tissues. A potential use of MSCs in the context of radiation-induced tissue lesions is supported by the radioresistant phenotype of this cell type. This review summarizes the current knowledge on the radiobiological features of these stem cells and highlights potential applications and challenges regarding the MSC-based repair of radiation damage.

## CELLULAR EFFECTS OF IONIZING RADIATION

### DNA damage signaling

**Figure 1:**
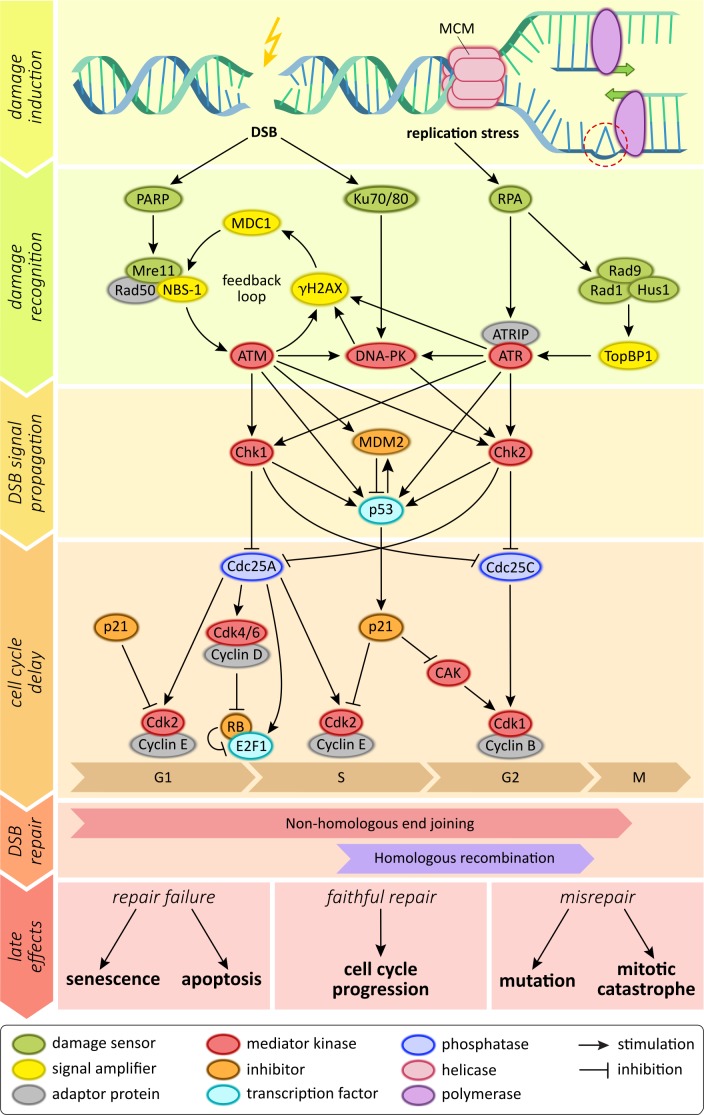
Schematic depiction of signaling molecules and pathways involved in the sensing of DNA double strand breaks

Ionizing radiation exerts its effects mostly on the cells' genomic information, either by directly depositing its energy onto DNA molecules or by creating free radicals that in turn interact with the DNA strands [36]. Depending on the energy transfer, irradiation creates various different forms of DNA damage, including damage to DNA bases or the sugar backbone as well as complex, clustered strand breaks that contain different DNA lesions with one region of the DNA [37]. While base damage or single-strand breaks occur much more frequently, DNA double strand breaks are considered the main toxic lesion by which ionizing radiation kills cells. Swift recognition and repair of DNA damage is crucial for affected cells; failure to repair may result in cell death, and misrepair may lead to an accumulation of mutations and genomic instability [38]. Therefore, most cells employ an intricate DNA damage signaling network (Figure 1). Within this network, the ataxia teleangiectasia mutated (ATM) serine/threonine kinase is one of the central regulatory proteins [39, 40]. It is recruited to sites of DNA damage by the Mre11-Rad50-Nbs1 DNA-binding complex, and phosphorylates various downstream components, including Chk2 protein and the histone variant H2AX [41]. These factors in turn recruit other downstream factors, resulting in massive signal amplification and finally in the recruitment of the components of key DNA repair pathways [39].

### DNA double strand break repair

**Figure 2:**
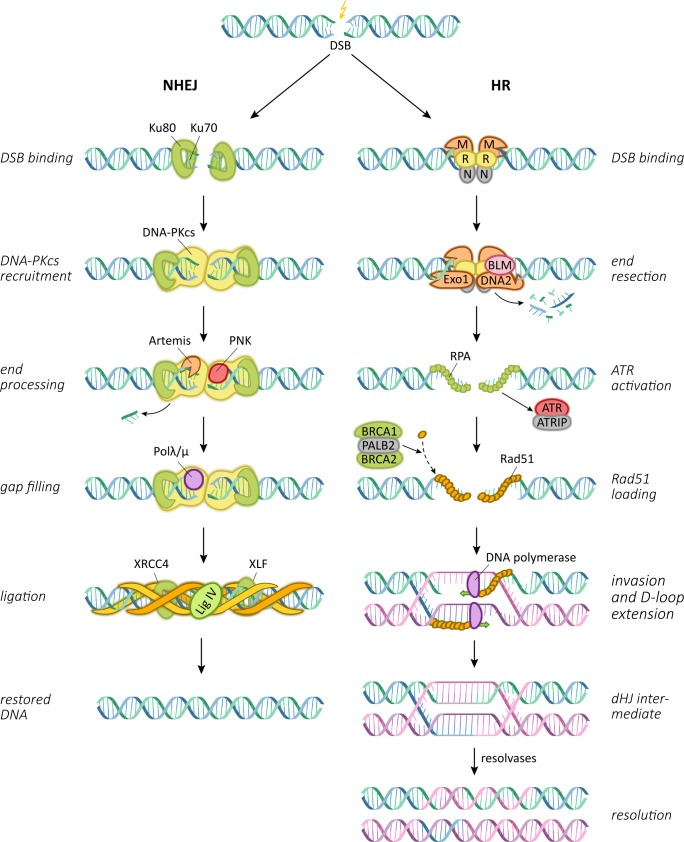
Central pathways and proteins involved in the repair of radiation-induced DNA double strand breaks NHEJ: non-homologous end joining; DSB: double strand break; HR: homologous recombination; dHJ: double Holliday junction.

Upon irradiation, DNA double strand breaks occur either directly or as a consequence of two closely located SSBs on opposite strands. As they may lead to the loss of crucial genomic information, their quick and efficient repair is important for cellular integrity and survival. Cellular repair of DNA double strand breaks is carried out by two major pathways, termed non-homologous end joining (NHEJ) and homologous recombination (HR) (Figure 2) [38, 42]. HR requires the presence of a sister chromatid and can therefore only take place during late S and G2 phases of the cell cycle; therefore, the majority of DNA double strand break repair is commonly carried out by the NHEJ pathway [43]. As an initial step of NHEJ, the strand break is recognized and labeled by the heterodimeric Ku protein complex [44, 45]. Ku in turn binds and thereby recruits the DNA-dependent protein kinase catalytic subunit (DNA-PKcs) to sites of double strand breaks [46]. DNA-PKcs has weak kinase activity and upon recruitment, can autophosphorylate and also phosphorylate a variety of other repair factors [47]. Clean double strand breaks without overhangs or modified strand ends can then be ligated and are accurately repaired by the NHEJ pathway [42]. However, break ends with additional radiation-induced modifications usually require end processing prior to re-ligation in order to remove other forms of DNA lesions and non-ligatable groups. In a last step, gaps that are created by end processing are filled in by DNA polymerases μ and λ, before the X4L4 protein complex consisting of DNA ligase IV and XRCC4 carries out the ligation step with the support of additional proteins like Cernunnos [42, 48-50]. Depending on the amount of additional damage or modifications at strand ends, removal of damaged sequences during NHEJ may result in the loss of a significant amount of genetic material and therefore often results in error-prone repair.

In contrast, the HR repair pathway carries out conservative DNA double strand break repair by using the sister chromatid as a template. The initial sensing of the strand break as a prerequisite for HR requires the proteins Mre11, Rad50 and Nbs1 that form a functional unit termed the MRN complex [51-53]. These MRN complexes assemble at both strand ends and recruit the key serine/threonine protein kinase Ataxia teleangiectasia mutated (ATM) which in turn phosphorylates multiple downstream proteins involved in the double strand break repair [54]. Similar to NHEJ, strand end processing is carried out during HR by several proteins including Exo1, BLM and BRCA1, and single strand ends are created [55-57]. The strand ends are then coated by Rad51 protein multimers which are believed to help detecting areas of homology on the sister chromatid [58, 59]. The Rad51-coated strand then invades the sister chromatid, and strand elongation is performed by DNA polymerases η or δ from the 3′ end [60, 61]. Upon completion of the template-based strand elongation, the repaired strand is either displaced from the duplex DNA and re-annealed, or requires resolution, as the second broken strand may also be captured by the homologous sequence, forming a double Holliday junction [62]. The final ligation is then performed by DNA ligase I [63].

## INFLUENCE OF IONIZING RADIATION ON MSCS

The radiation response of MSCs has been analyzed both in mouse and human cell samples; several studies using high-dose irradiation have demonstrated that the in-vitro survival of MSCs was considerably higher than that of other bone marrow-derived stem cells and was similar to other rather radio-resistant cell lines [64-66]. Another analysis demonstrated increased radiation resistance of adult human MSCs compared to embryonic stem cells [67]. The described radio-resistance was found to be even more pronounced when MSCs were exposed to fractionated or even hyperfractionated courses of irradiation with single doses ranging between 0.5 and 2 Gy [68, 69]. Exposure to low-dose radiation was reported to result in increased proliferation of cultured rat MSCs, and even radiation doses as low as 0.01 Gy were found to influence expression patterns of genes involved in DNA replication, stress response, DNA repair and translation initiation [70, 71]. The reported relative radiation resistance of MSCs was found more pronounced in hypoxic conditions with hypoxic mouse MSCs showing increased proliferation, increased DNA damage repair and improved long-term survival after exposure to ionizing radiation [72]. Similar to the reported in-vivo data, the radioresistance of MSC has also been shown *in vivo*: MSCs extracted from porcine mandibular bones after irradiation with single doses up to 18 Gy retained their ability to proliferate and were still able to differentiate along the osteogenic and adipogenic lineages [73].

While most of the published data examined the effects of conventional photon radiotherapy on the stem cells, only few data are available on other forms of ionizing radiation, including proton or heavy-ion treatments. Understanding particle radiobiology becomes more and more important because ions such as protons and carbon ions are increasingly being studied in clinical facilities worldwide for the radiotherapy of cancers, but also in the context of manned space flights, as particle radiation is highly prevalent in deep space and remains a major obstacle for space flights. Experiments using ion radiation in MSCs showed that doses of ^56^Fe ions up to 1 Gy were found to lead to more pronounced G2/M phase arrest than physically equivalent photon doses [74]. Another study examining biologically equivalent doses showed that MSCs were similarly resistant to high doses of ^12^C carbon ion and photon irradiation and retained their stem cell features after both forms of ionizing radiation [75].

The defining stem cell properties of MSCs were shown to be largely intact after exposure to ionizing radiation. The ability of MSCs to swiftly adhere to plastic surfaces and migrate appeared unaffected, and the characteristic adipogenic, osteogenic and chondrogenic differentiation potential could not be abolished by ionizing radiation, although there have been suggestions about a dose-dependent reduction at very high single doses exceeding 10 Gy [65, 76, 77]. Conversely, the differentiation level was demonstrated to influence the radiation response of MSCs, albeit with somewhat contradictory findings: While differentiation along the adipogenic and osteogenic lineages was found to render MSCs more sensitive to ionizing radiation, another study showed that differentiation-restricted, high passage-number cells seemed to be more resistant compared to their pluripotent, low passage-number counterparts [78, 79].

### Activation of DNA damage recognition pathways in MSCs

Various publications analyzed potential mechanisms underlying the relative radio-resistance of MSCs and have suggested a strong influence of the known DNA damage recognition pathways. As described above, the serine/threonine kinase ATM is a crucial factor responsible for the recruitment and activation of multiple downstream DNA damage sensing and repair proteins and the initiation of the HR repair pathway; deficiency in ATM function results in increased radiosensitivity [80, 81]. The importance of ATM signaling for the radiation response of MSCs was underlined by several studies (Table 1): Expression analyses in mouse stem cell samples found high constitutive expression levels of ATM and the downstream kinase Chk2 with and without radiation treatment [12]. While ATM expression was reported to be stable even after very high radiation doses of up to 60 Gy, irradiation of MSCs both with low and high photon doses and ^12^C particles was observed to strongly increase ATM autophosphorylation and activation [75, 82, 83]. However, small-molecule inhibition of ATM in MSCs did not abrogate accumulation in G2 phase of the cell cycle, suggesting compensatory pathways, likely involving the key NHEJ kinase DNA-PKcs [84]. Several regulatory proteins have been observed to be phosphorylated in an ATM-dependent fashion in MSCs, including p53, Chk2 and replication protein A [79, 82, 84]. Knockdown of the ATM-dependent downstream kinases Chk1 and Chk2 was reported to increase radiosensitivity of MSCs as measured by residual DNA double strand breaks and increased levels of apoptosis [85]. Besides the ATM pathway, the function of DNA-PKcs was found to be comparably important for the DNA damage signaling of MSCs, and prolonged phosphorylation of DNA-PKcs was observed in stem cells with unrepaired DNA double strand breaks [79, 82]. The damage signaling patterns reported in MSCs suggest that both DNA double strand break repair pathways play an important role in the radiation response of these cells.

**Table 1:** The influence of irradiation on key components of the DNA damage signaling and repair pathways in mesenchymal stem cells

Protien	Function in irradiated MSCs	References
ATM	High baseline levels	[12]
	Strong autophosphorylation after photon irradiation	[75, 82, 83]
	Strong autophosphorylation after 12C irradiation	[75]
	Strong phosphorylation of downstream targets Chk2, p53 and RPA	[79, 82, 84]
	Regulation of G2 cell cycle arrest	[12, 65, 75, 86]
Chk2	Increased phosphorylation	[12]
	Increased phosphorylation	[79, 82]
DNA-PKcs	Strong autophosphorylation	[79, 82]
Ku70	Increase of nuclear levels	[79]
P53	Strong induction	[82]
	Stabilization	[87]
P21	Strong induction	[82]
	Prolonged expression	[88]

Additionally, expression analyses in MSCs reported a strong induction of the cell cycle-regulating proteins p53 and p21 by ionizing radiation [82]. Activation of the damage signaling pathways was found to result in transient delays in G1/S, S or G2/M phases of the cell cycle. It has been shown that the ATM-dependent pathway activation was able to induce cell cycle arrests both in mouse and in human MSCs. While the observed effects in mouse cell lines were weak and transient, the arrest described in human samples was more pronounced and appeared both in G1/M and G2 phases [12, 65, 86]. This observation has been attributed to a stabilization of p53 and prolonged expression of the downstream protein p21 in irradiated human MSCs [87, 88].

### DNA damage repair capacities of MSCs

As outlined above, DNA double strand breaks as the critical radiation-induced lesions are commonly repaired by the HR and NHEJ pathways, dependent on the cellular repair capacity and the cell cycle phase. Several studies analyzed the activity of DNA double strand break repair in MSCs and found efficient repair of these lesions after irradiation as measured by phosphorylated histone H2AX (γH2AX) levels [79, 82, 87, 89]. Even following high doses of photon or ^12^C particle radiation, MSCs were found to efficiently repair DNA double strand breaks within 24 hours [75]. This swift repair seems to be mediated by both the NHEJ and the HR pathways: Upon exposure to ionizing radiation, the quick but potentially error-prone NHEJ pathway was found to be strongly activated in MSCs; and blockade of the NHEJ pathway by chemical inhibition of DNA-PKcs reportedly resulted in lower numbers of initial γH2AX foci but increased residual levels at 24 hours, suggesting a higher ratio of unrepaired double strand breaks [64, 82]. Additionally, nuclear Ku70 levels were found to increase in MSCs after irradiation, an effect that was even more pronounced when MSCs underwent adipogenic or osteogenic differentiation [79]. While the NHEJ nuclease DCLRE1C/Artemis was shown to prevent genomic damage in MSCs after exposure to ionizing radiation, it seemed dispensable for the radiation-dependent survival of MSCs, and DCLRE1C-deficient cells were found to be even more radioresistant than their functional counterparts [90]. In this context it has been proposed that a deficiency in the NHEJ pathway may introduce a “paraneoplastic” MSC phenotype.

Besides NHEJ repair, the dependence of MSCs on the HR pathway for the repair of radiation-induced double strand breaks has also been demonstrated. Inhibition of ATM in irradiated MSCs resulted in increased residual H2AX foci levels, demonstrating a dependence of these stem cells on the initiation of HR [82]. However, the ATM blockade did not abrogate H2AX phosphorylation or cell cycle arrest, suggesting an interplay between HR and NHEJ in irradiated MSCs [84]. Experiments using mouse MSCs deficient in the Fanconi anemia group protein FANCD2 showed that those cells were more radiosensitive to ionizing radiation, underpinning the importance of homology-directed double strand break repair [91]. Hypoxic MSCs were found to be even more proficient in the HR repair of radiation-induced DNA double strand breaks, as Rad51 was shown to be regulated by the hypoxia-inducible factor 1α (HIF1α) [72, 92].

### The effects of ionizing radiation on MSC apoptosis, senescence and autophagy

Upon induction of irreparable DNA damage by ionizing radiation, cellular mechanisms such as apoptosis, senescence or autophagy have been described for a variety of cell types. Several studies have analyzed the induction of apoptosis in MSCs after irradiation. Doses up to 20 Gy were observed to only result in minimal induction of cellular apoptosis [64, 65, 75, 84, 86, 93]. These findings were supported by expression analyses of MSCs, demonstrating high expression levels of anti-apoptotic proteins BCL-2 and BCL-XL, while only low levels of pro-apoptotic proteins such as Puma were reported for these cells [12, 87].

In contrast, cellular senescence has been shown to strongly increase upon exposure of MSCs to ionizing radiation and has been hypothesized to be the main coping mechanism by which MSCs avoid proliferation after severe genomic damage [84, 94]. Radioresistant MSCs populations were found to exhibit both strong β-galactosidase activity and an increased expression of the cyclin-dependent kinase inhibitor 2A (p16-INK4A) at late time points after irradiation with doses up to 60 Gy [83, 84]. Additionally, treatment with low radiation doses between 0.04 Gy and 2 Gy was observed to result in increased levels of senescence without affecting apoptosis [93]. Induction of senescence in MSCs was found to be mediated by both the retinoblastoma protein, pRB, and the tumor suppressor, p53 [95, 96]. The senescence of MSCs induced by high doses of IR resulted in reduced osteogenic and adipogenic differentiation potentials [96]. Additionally, the reorganization of the MSC cytoskeletal architecture as regulated by the protein kinase CK2 has been suggested as a result of radiation-induce senescence, although other publications did not find an influence of irradiation on the MSC cytoskeleton [65, 97]. On a molecular level, typical senescence-associated DNA methylation changes were found to be missing in irradiated MSCs, suggesting a potential evasion mechanism contributing to the reported radiation resistance of these cells [98].

To date, there is only little evidence for the role of autophagy in irradiated MSCs, and the published data are somewhat contradictory. One study reported a reduction in the autophagy levels after both high and low-dose irradiation, suggesting a decreased ability to remove damaged cellular components [99]. Other publications have discussed autophagy as another means by which MSCs prevent radiation injury. The induction of autophagy in MSCs was shown to lead to reduced levels of radiation-induced reactive oxygen species (ROS), resulting in the preservation of the cellular differentiation potential and the expression of stem cell markers [100, 101]. Autophagy was observed to be increased in MSCs by hypoxia via activation of the ERK1/2 and mTOR pathways, linking with previous findings that hypoxia could increase MSC radioresistance [72, 102, 103].

### Heterogeneity of the MSC radiation resistance

MSCs seem to comprise a heterogeneous cellular population and are therefore often termed multipotent mesenchymal stromal cells to differentiate them from other clonal stem cells of the bone marrow. Comparative analyses reported varying levels of radioresistance in human and mouse MSCs derived from different organ sites [86, 104]. Additionally, several publications have suggested that each MSC pool contains only a certain subpopulation with stem cell characteristics which defines the stem cell-like properties of this cell type rather than the whole pool forming a stem cell population [3, 105-108]. Therefore, it has been proposed that the observed radioresistant phenotype may also be due to a radioresistant subpopulation of MSCs, as has been reported for cancer-initiating cells. However, only limited experimental data back up this hypothesis [83, 99]. Data published on the effects of high-dose radiation with 30 and 60 Gy on MSCs identified a highly radioresistant MSC subpopulation within the tested cell pool that not only survived the treatment, but also kept the ability for tri-lineage differentiation [83]. The characterized MSCs were found to keep their surface marker expression but had altered secretion profiles of VEGF and PDGF and became prematurely senescent. In this context, the level of “stemness” and the role of certain MSCs as precursors of other partially restricted MSCs have been discussed [87, 104]. However, several other publications report strong radioresistance effects from pooled MSC populations, and those findings are difficult to be attributed to only a small sub-group of these cells [12, 64, 65]. Therefore, further efforts are needed to clearly identify radioresistant subpopulations within the MSC population.

## POTENTIAL APPLICATIONS AND CHALLENGES OF RADIORESISTANT MSCS

### Repair of radiation-induced tissue damage

The role of MSCs in the repair of various forms of tissue damage has been well established both *in vitro* and in animal models, especially in the context of mechanical and ischemic lesions [34, 35, 109-114]. Regarding the demonstrated radiation resistance of MSCs, these cells may also have a role in the regeneration of organ damage induced by ionizing radiation. Apart from the involvement of the body's own MSCs in the repair of radiation damage that remains largely elusive, potential benefits of both treatment with autologous MSCs and allogeneic infusions of MSCs have been widely studied *in vitro* and in animal models. MSC infusions after irradiation of different organs with lethal or sub-lethal doses resulted in improved overall survival in several datasets [115-120]. However, it has been suggested that beneficial effects of MSC-based treatments were dependent on the timing of infusions and cell numbers [121]. The exact underlying mechanisms for the MSCs' radioprotective effects are yet to be established. Radiotherapy was widely found to induce both the homing and the engraftment of transplanted MSCs in radiation-damaged organs in rat and mouse models [122-127]. Further examinations demonstrated detectable levels of allogeneic GFP-labeled MSCs in radiation-damaged tissue samples up to 80 days after infusion [128]. However, other analyses reported only transient invasion of MSCs, and it has been suggested that their beneficial effect may be attributable to paracrine effects or the programming of other involved cell types [129]. This finding has been backed up by the observation that treatment of irradiated animals with MSC-conditioned media mimicked the increased repair of radiation damage [130]. Generally, the generation of clear data regarding mechanistic aspects of MSC-mediated repair of tissue radiation damage has been somewhat hampered by the lack of generally accepted surface markers, which would help to detect MSCs in damaged tissues and clearly attribute observed effects to these cells [7, 87]. Beyond the data generated from animal models, first MSC-based therapies in humans treated for acute radiation injuries, mostly on the grounds of compassionate use, have revealed clinical benefits regarding the regeneration of hematopoiesis and radiation-induced intestinal inflammation and hemorrhages [131, 132]. Additionally, a Russian trial of 11 patients treated for radiation-induced pulmonary damage showed moderate improvements of breathing parameters, immune functions and tissue hypoxia by MSC infusions [133]. However, stringent clinical trials are required to properly establish potential clinical benefits of MSC-based treatments for radiation injuries. Additionally, the role of the body's own stem cells in the repair of radiation-induced and the development of radiation side effects remains largely unknown. Nevertheless, if MSCs are verified to attribute to the regeneration of radiation-induced tissue damage, their potential impact may affect diverse fields including medical procedures, radiation protection efforts and space flight endeavors. Beyond the alleviation of radiation treatment-related side effects, MSCs may have the potential to evolve as a means to support radiation protection efforts in the event of a nuclear radiation disaster or in the context of tackling the cosmic radiation risk for space flights, where large experimental programs are currently underway to study their biological effects [134-137].

### Radioprotection of tumor tissues

As outlined above, MSCs have been accredited with exhibiting radioprotective and regenerative functions in normal tissues exposed to ionizing radiation. Therefore, it is possible that they may similarly exert their effects on tumors treated with radiotherapy. However, there is a lack of published in-vivo data regarding the influence of MSCs on the radioprotection or regeneration of cancer tissue exposed to ionizing radiation. In an in-vitro study, breast cancer cells were found to exhibit increased proliferation rates and radioresistance by treatment with MSC-conditioned medium, an effect attributed to the high levels of insulin-like growth factor-1 within the medium [138]. Despite the fact that no in-vivo data have been published to back up this finding, there is still a potential risk that radiotherapy itself increases the radiation resistance of cancer tissues by increasing the attraction and engraftment of MSCs into the irradiated tumor microenvironment. Data obtained from irradiated mice demonstrated that low dose radiation treatment resulted in increased invasion of MSCs from the perivascular into the parenchymal areas of tumors and increased secretion of the cytokines, PDGF, VEGF and TGFβ [139]. Additionally, several publications have shown the angiogenic potential of MSC-based treatments, and MSC infusions were found to increase angiogenesis in ischemic tissues [140-144]. The impact of the MSCs' angiogenic properties on the tumor response to radiotherapy has been subject to some discussions. While it may be possible that these stem cells can counteract antiangiogenic tumor therapies, it is likewise conceivable that they may improve the oxygen supply to tumor tissues, thereby increasing the radiosensitivity of irradiated cancers. It has been shown that MSC proliferation is dependent on proangiogenic signaling molecules such as VEGF, and VEGF inhibition severely radiosensitized MSCs *in vitro* [66]. While it can be speculated that MSCs may exert some radioprotective effects on tumors cells, in-vivo data are lacking. However, if these speculations are backed up by experimental findings, the dose-dependent increase in intratumoral migration of MSCs may pose additional problems due to the fact that MSCs themselves are radioresistant and may not be properly targeted by the radiation treatment. Co-culture data provided some evidence for the creation of an inflammatory microenvironment, resulting in an impaired sensitivity to anti-cancer treatments and ultimately in the promotion of tumor growth and an increased risk of tumor recurrence [145]. To rule out potential radioprotective effects of MSCs on irradiated tumors, solid in-vivo data are needed that take into account the effects of MSCs on the microenvironment and their interaction with other cell types of the tumor stroma. These data are a prerequisite for establishing any form of MSC-based therapy for radiation-induced tissue damage in the clinical routine.

## SUMMARY

The influence of ionizing radiation on adult mesenchymal stem cells has been extensively examined. It has been well established that MSCs exhibit a radioresistant phenotype and are able to retain their defining stem cell properties after exposure to high radiation doses. The published research indicates that MSCs are able to quickly and efficiently recognize radiation-induced DNA damage via different recognition pathways. Additionally, a strong activity of both DNA double strand break repair pathways, homologous recombination and non-homologous end joining, has been linked to the described radioresistant phenotype of MSCs. High expression levels of anti-apoptotic proteins result in the cells' evasion of apoptosis even after high doses of ionizing radiation, while the role of senescence and autophagy requires further examination. Due to their radioresistant phenotype, MSCs may qualify as a therapeutic means to treat radiation-induced tissue damage. However, potential protective effects on irradiated tumors require further research into this cell type, before MSC-based therapies will find their way into the clinic for the treatment of radiation-induced tissue damage.
